# Biodegradable Polymeric Nanocapsules Prevent Cardiotoxicity of Anti-Trypanosomal Lychnopholide

**DOI:** 10.1038/srep44998

**Published:** 2017-03-28

**Authors:** Renata Tupinambá Branquinho, Jérôme Roy, Charlotte Farah, Giani Martins Garcia, Franck Aimond, Jean-Yves Le Guennec, Dênia Antunes Saude-Guimarães, Andrea Grabe-Guimaraes, Vanessa Carla Furtado Mosqueira, Marta de Lana, Sylvain Richard

**Affiliations:** 1 Pharmaceutical Sciences Post-graduation Program (CiPharma), Escola de Farmácia, Universidade Federal de Ouro Preto, Minas Gerais, Brazil; 2 PHYMEDEXP, Inserm U1046, CNRS UMR 9214, Université de Montpellier – Montpellier, France

## Abstract

Chagas disease is a neglected parasitic disease caused by the protozoan *Trypanosoma cruzi*. New antitrypanosomal options are desirable to prevent complications, including a high rate of cardiomyopathy. Recently, a natural substance, lychnopholide, has shown therapeutic potential, especially when encapsulated in biodegradable polymeric nanocapsules. However, little is known regarding possible adverse effects of lychnopholide. Here we show that repeated-dose intravenous administration of free lychnopholide (2.0 mg/kg/day) for 20 days caused cardiopathy and mortality in healthy C57BL/6 mice. Echocardiography revealed concentric left ventricular hypertrophy with preserved ejection fraction, diastolic dysfunction and chamber dilatation at end-stage. Single cardiomyocytes presented altered contractility and Ca^2+^ handling, with spontaneous Ca^2+^ waves in diastole. Acute *in vitro* lychnopholide application on cardiomyocytes from healthy mice also induced Ca^2+^ handling alterations with abnormal RyR2-mediated diastolic Ca^2+^ release. Strikingly, the encapsulation of lychnopholide prevented the cardiac alterations induced *in vivo* by the free form repeated doses. Nanocapsules alone had no adverse cardiac effects. Altogether, our data establish lychnopholide presented in nanocapsule form more firmly as a promising new drug candidate to cure Chagas disease with minimal cardiotoxicity. Our study also highlights the potential of nanotechnology not only to improve the efficacy of a drug but also to protect against its adverse effects.

Chagas disease (CD) is an important neglected disease caused by an intracellular hemoflagellate protozoan parasite, *Trypanosoma cruzi (T. cruzi)*. There are an estimated 6–7 million infected people worldwide, and the disease causes 12 500 deaths per year, mostly in Latin America (World Health Organization, Switzerland 2015). While CD is principally transmitted to humans by triatomine insects, other routes include blood transfusion or transplantation of contaminated organs, ingestion of contaminated food and congenital transmission from infected mothers to newborns. International migrations contribute to spread the disease in non-endemic areas such as North America, Europe and Western Pacific countries (World Health Organization, Switzerland 2015). CD evolves in different consecutive phases with a short acute period, characterized by patent parasitemia, followed by a chronic phase in which most infected individuals remain asymptomatic (indeterminate form). CD is also a common determinant of non-ischemic cardiomyopathy in addition to digestive complications and neurological disorders. Approximately 30% of patients infected with *T. cruzi* manifest cardiomyopathy characterized by severe myocarditis and multiple arrhythmias, and/or dilatation and physiological dysfunctions of hollow organs, mainly of the digestive tract, which develop progressively over the years or decades after infection^1^,^2^,^3^,^4^. The clinical course of CD shows great variability, and the mechanisms or determinants responsible for the development of cardiomyopathy are unclear^5^.

Although vector control helps to prevent CD transmission in endemic areas, pharmacological treatment is essential to cure the disease in infected people. Therapeutic options to kill the parasite are currently limited to benznidazole and nifurtimox. However, while treatment during the early stages of CD is effective, treatment during the chronic phase has been relatively ineffective^6^,^7^. In addition to their limited efficacy against the complications of CD during chronic infection, the two available drugs often have shortcomings related to high toxicity, drug resistance of some *T. cruzi* strains or genetic groups of the parasite, long treatment regimens, which reduce patient compliance^8^. There is no safe drug available to cure patients at chronic phase of the Chagas disease. Among the important issues regarding the risk/benefit ratio of the existing drugs are their adverse effects in a weakened population with potential cardiac complications during the chronic phase of the disease^9^. The identification of new chemical entities or pharmaceutical formulations capable of effectively and reliably killing the parasite, not only in the acute but also in the chronic phase before irreversible heart damage in infected individuals, and that, moreover, have minimal side effects, is thus an urgent necessity^10^.

Recently, we have documented the antitrypanosomal efficacy *in vivo* of a natural substance, lychnopholide (LYC), isolated from *Lychnophora trichocarpha* Spreng, a plant from *Asteraceae* family. In particular, a pharmaceutical formulation of LYC encapsulated in biodegradable polymeric nanocapsules (NC) and administered by intravenous route was more effective than benznidazole in curing Swiss mice infected experimentally with different *T. cruzi* strains^11^,^12^. The LYC associated to NC improved drastically the free LYC efficacy after intravenous administration. LYC NC was also effective in the chronic phase of the infection in mice model^12^, consistent with a probable improvement of the pharmacokinetic profile of LYC to reach the parasites in tissues, as reported (Branquinho *et al*., submitted *Scientific Reports*). Pegylated biodegradable NC (PLA-PEG) increased 26-fold the body exposure to LYC and increased the LYC plasma half-life 25-times compared to free LYC solution administered intravenously. These results are in line with efficacy data upon *iv* treatment with NC, 100% *versus* 0% mice cure for NC and free LYC, respectively. At the best of our knowledge, no other drug has been so efficient to cure experimental *T. cruzi* infection at chronic phase in mice. However, no safety aspects related to the use of LYC *in vivo* have been investigated to date. As the infection induces serious cardiac alterations, the LYC cardiotoxicity evaluation in healthy mice is of primordial importance in the first investigation of LYC safety. Another point is the ability of NC to increase and maintain plasma levels of LYC, which may enhance toxicity as a consequence. Cardiotoxicity is the first cause of a new drug candidate to be abandoned in the pre-clinical phase. We undertook this study with a three-fold objective: (i) to identify and investigate any potential toxicity of LYC on cardiomyocytes *in vitro*; (ii) to study *in vivo* cardio-toxicological effects of repeated LYC intravenous administration on cardiac function, and, (iii) to study the *in vivo* effect of the encapsulation of LYC in NC. Our main results show that repeated dose treatment with LYC for 20 days (2 mg/kg/day), which had previously demonstrated anti-trypanosomal efficacy in mice, could induce severe cardiopathy with 50% mortality in healthy mice and alterations of Ca^2+^ handling in single cardiomyocytes. Dose-dependent alterations of Ca^2+^ handling, promotion of Ca^2+^ waves, and abnormal RyR2-mediated diastolic Ca^2+^ release were also observed following acute application of the drug. Strikingly, all adverse cardiac effects were prevented when LYC was administered associated to the polymeric biodegradable NC.

## Results

### NC encapsulation prevented mortality and heart failure induced by repeated-dose LYC treatment

**Figure 1:**
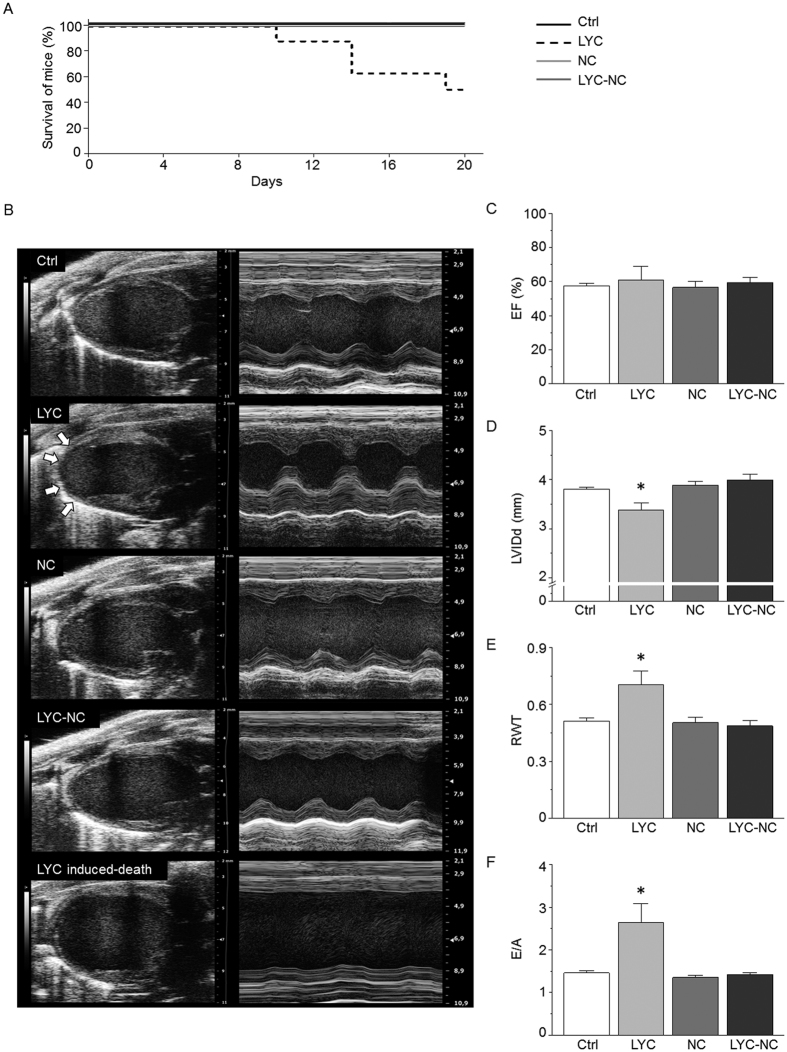
NC prevented mortality and heart failure triggered by repeated-dose administration of free LYC. (**A**) Survival curves of mice treated for 20 days. Groups were Ctrl (vehicle, 10 mice), lychnopholide (free LYC 2.0 mg/kg/day; 8 mice), unloaded biodegradable polymeric nanocapsules (NC; 10 mice) and lychnopholide loaded in polymeric nanocapsules (LYC-NC, 10 mice). Only mice from the LYC group died (p = 0.0005; Log-rank (Mantel-Cox test). (**B**) Illustrations of transthoracic echocardiography performed at day 19 for each group, excepted for one mouse from the LYC group (bottom panels) examined at day 18 because of critical heart status and before sudden death at day 19. Left panels: left ventricular parasternal long-axis B-mode views in diastole. Arrows show ballooned apex observed in LYC mice. Right panel: left ventricular parasternal long-axis M-mode views. (**C–F**) Averaged effects for each experimental group on left ventricular ejection fraction (EF) assessed in parasternal long-axis view by the Teicholz method (**C**), left ventricular internal diameter in diastole (LVIDd) (**D**), relative wall thickness (RWT) (**E**), ratio of the peak velocity of the early left ventricular filling wave (**E**) to the late atrial contraction left ventricular filling wave (**A**), used as an index of left ventricular diastolic function (**F**). *p < 0.05, LYC *vs*. other groups.

The mean hydrodynamic diameters of blank NC and LYC-NC were 110.1 ± 2.2 nm and 105.1 ± 4.4 nm, respectively. These colloidal formulations were monodispersed in size with polydispersity indexes lower than 0.3 indicating uniformity in the nanoparticle population. Detailed LYC long-circulating NC development and characterization was reported elsewhere (back-to-back paper submitted to Sci. reports). LYC and the LYC-NC formulation were administered *in vivo* at the dose found to be effective in killing *T. cruzi* in the acute and chronic phases of infection in mice by intravenous (2 mg/kg/day) administration routes^12^. Here, repeated intravenous administration of free LYC (2.0 mg/kg once a day) for 20 days induced 50% mortality in C57BL/6 mice as shown by the survival curve (Fig. 1A). The encapsulation of LYC completely prevented mortality, since no mice died in the LYC-NC group. Heart morphology and function were evaluated by echocardiography in surviving mice one day before sacrifice (day 20) (Fig. 1B). Neither LYC-NC nor blank NC affected the ejection fraction (EF) (Fig. 1C). However, free LYC induced concentric myocardial hypertrophic remodelling characterized by increased left ventricular (LV) wall thickness (Fig. 1B; see Suppl. Table 1) and decreased LV diameter in diastole (Fig. 1D), leading to a higher relative wall thickness (RWT) when compared to the intravenous control (Ctrl) group (Fig. 1E). In contrast, heart morphology and function were unaffected by administration of blank NC (vs. Ctrl group; Fig. 1B–E). Importantly, the administration of LYC encapsulated in NC prevented the adverse effects of LYC. There was no difference between the LYC-NC group and the Ctrl group in any functional parameter measured (Fig. 1B–E; see Suppl. Table 1), in line with the idea that the encapsulation of LYC prevents the deleterious remodelling associated with LYC in its free form. Interestingly, LYC-treated-mice exhibited a ballooned apex reminiscent of Tako-tsubo cardiomyopathy^13^,^14^. In light of this particular type of hypertrophic wall remodelling and the dilated apex observed in the LYC group, we also assessed the EF by the Simpson method to circumvent the limitations of the Teicholz method. No difference was observed between the groups (see Suppl. Table 1). Surprisingly, the main LV remodelling induced by LYC was an increase of the E/A ratio (LYC vs. Ctrl; Fig. 1F), which indicated that the myocardium did not properly fill up with blood between two contractions, highlighting a diastolic dysfunction. This dramatic effect was also prevented by the encapsulation of LYC. In addition, in line with both the hypertrophy and diastolic dysfunction observed, the LYC group was characterized by an increase in the isovolumetric relaxation time, a decrease in the mitral valve deceleration velocity and an increase in the E/E′ ratio (see Suppl. Table 1), altogether reflecting higher LV filling pressure and a restrictive mitral wave profile. Also consistent with these alterations, we observed a higher left atrium diameter (LYC *vs.* Ctrl and NC groups), suggesting adaptive remodelling to compensate for the LV diastolic dysfunction. Finally, because of a critical phenotype, one of the free LYC-treated mice underwent echocardiographic at day 18, and died suddenly at day 19. This mouse presented a massively dilated LV (bottom panel of Fig. 1B) consistent with a brutal progression from hypertrophy to decompensated heart failure (HF) induced by LYC. Altogether, our results reveal the harmful and life-threatening cardiac effects of LYC during repeated-dose administration regimen. However, these effects were prevented by the encapsulation of LYC in polymeric NC in the same experimental conditions and dose regimen.

### NC preserved cell contractility and Ca^2+^ handling in mice during repeated-dose LYC treatment

**Figure 2:**
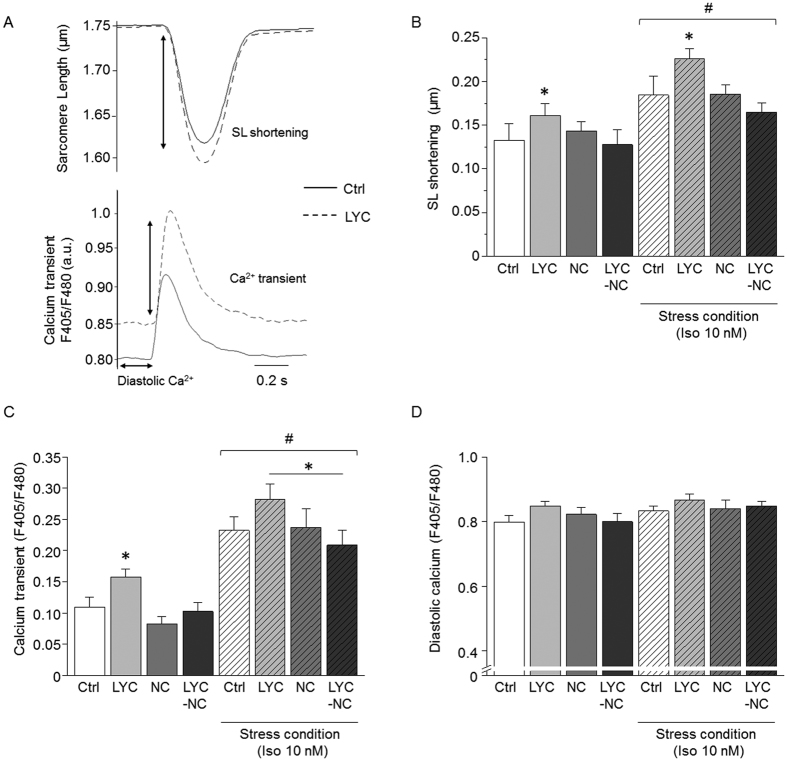
NC prevented alterations of cellular contractility and Ca^2+^ handling promoted by repeated-dose administration of free LYC. (**A**) Typical traces of sarcomere shortening (top panel) and Ca^2+^ transient (bottom panel) measured in single cardiomyocytes, field stimulated at 1 Hz, from control and LYC groups after 20 days of treatment. Sarcomere length (SL). (**B**–**D**) Averaged effects of 20 days of treatment for the Ctrl, LYC, NC and LYC-NC groups under basal and acute stress conditions (Iso 10 nM) on sarcomere shortening (**B**), Ca^2+^ transient amplitude (**C**) and diastolic Ca^2+^ level expressed as the ratio of fluorescence at 405 and 480 nm; a.u.: arbitrary units (**D**) *p < 0.05, LYC *vs*. other groups under the same conditions; ^#^p < 0.05, basal *vs*. stress; 10–23 cells per group.

We investigated the cellular phenotype associated with the LYC-induced cardiac remodelling revealed by echocardiography. The contractility of single cells and intracellular Ca^2+^ were measured in electrically stimulated cardiomyocytes (Fig. 2A). LYC increased sarcomere shortening, an index of cellular contractility, during stimulation (Fig. 2A,B). This positive inotropy was associated with increased Ca^2+^ transients as measured with the fluorescent ratiometric dye indo-1 (Fig. 2A,C) and variable effects on diastolic Ca^2+^ levels during pacing (Fig. 2A,D). In sharp contrast, the encapsulation of LYC in NC abolished the effects of free LYC on cell shortening (Fig. 2B) and Ca^2+^ transient amplitude (Fig. 2C) as compared to the Ctrl or the NC group which was also unaffected. We next challenged single cells by the acute application of the β-adrenergic receptor agonist isoproterenol (Iso; 10 nM), which is commonly used to mimic the effects of exercise and stress. As expected, Iso increased sarcomere shortening and Ca^2+^ transient amplitude in all experimental groups (Fig. 2B–D). In the presence of Iso, LYC essentially had the same effects as under non-stressed conditions, on sarcomere shortening (Fig. 2B), Ca^2+^ transient amplitude (Fig. 2C). Again, these effects were prevented by the encapsulation of LYC (LYC-NC group), as under non-stressed conditions (Fig. 2B–D).

### NC prevented the occurrence of Ca^2+^ waves during repeated-dose LYC treatment

**Figure 3:**
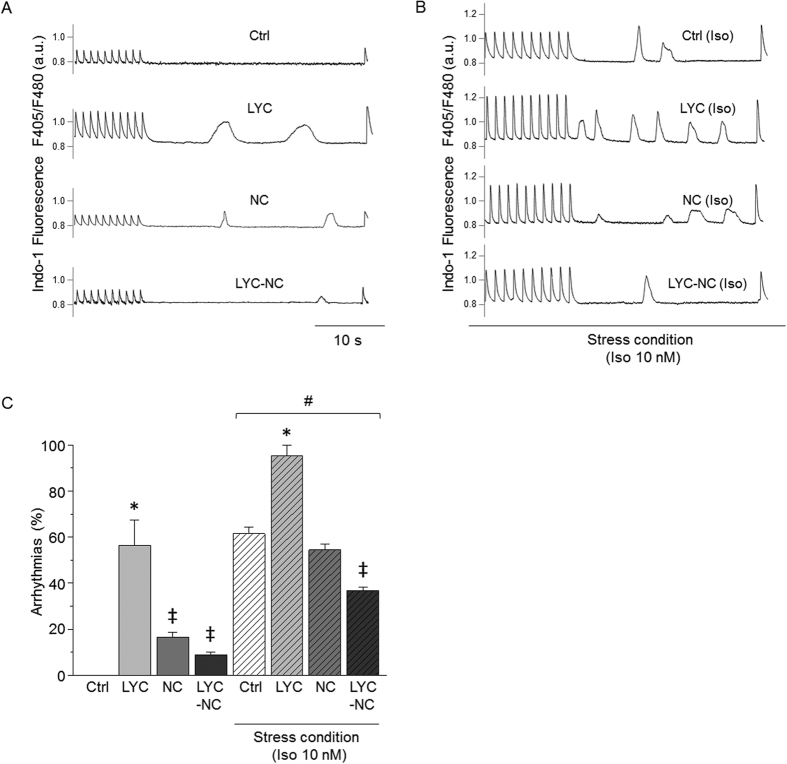
NC prevented abnormal spontaneous diastolic Ca^2+^ waves promoted in cardiomyocytes by repeated-dose administration of free LYC. (**A** and **B**) Representative traces of spontaneous Ca^2+^ waves during resting periods following a train of stimulation in cardiomyocytes from Ctrl, LYC, NC and LYC-NC groups after 20 days of treatment, under basal (**A**) and acute stress conditions (Iso 10 nM) (**B**). (**C**) Effect of 20 days of each treatment on the percentage of cardiomyocytes developing at least one spontaneous Ca^2+^ wave. *p < 0.05, LYC *vs*. other groups under the same conditions; ^‡^p < 0.05, *vs*. Ctrl under the same conditions; ^#^p < 0.05, basal *vs*. stress condition; 20–30 cells per group.

During the resting period following a train of stimulation, repeated-dose LYC treatment promoted a high occurrence of abnormal events, such as spontaneous Ca^2+^ waves (no event in Ctrl cells) under basal conditions (Fig. 3A,C). These Ca^2+^ waves were largely prevented by LYC encapsulation in NC, although blank NC alone slightly increased the triggering of Ca^2+^ waves. An acute β-adrenergic challenge with Iso highly favored basal Ca^2+^ waves even in the Ctrl group as well-known with this agent. However, this propensity was further aggravated in LYC-treated mice, in which nearly all cells exhibited diastolic Ca^2+^ events (Fig. 3B,C). Again, encapsulated LYC not only prevented the increased incidence of Ca^2+^ waves with free LYC, but also reduced the increased occurrence of Ca^2+^ waves in the presence of Iso alone in Ctrl animals. Altogether, our results showed that both under basal conditions and during Iso-induced stress, the repeated-dose administration of LYC modifies contractility, Ca^2+^ handling and the occurrence of abnormal spontaneous Ca^2+^ waves. Of major importance, these alterations were prevented by the encapsulation of LYC in polymeric NC.

### Acute effects of LYC on Ca^2+^ homeostasis and Ca^2+^ sparks in myocytes from healthy mice

**Figure 4:**
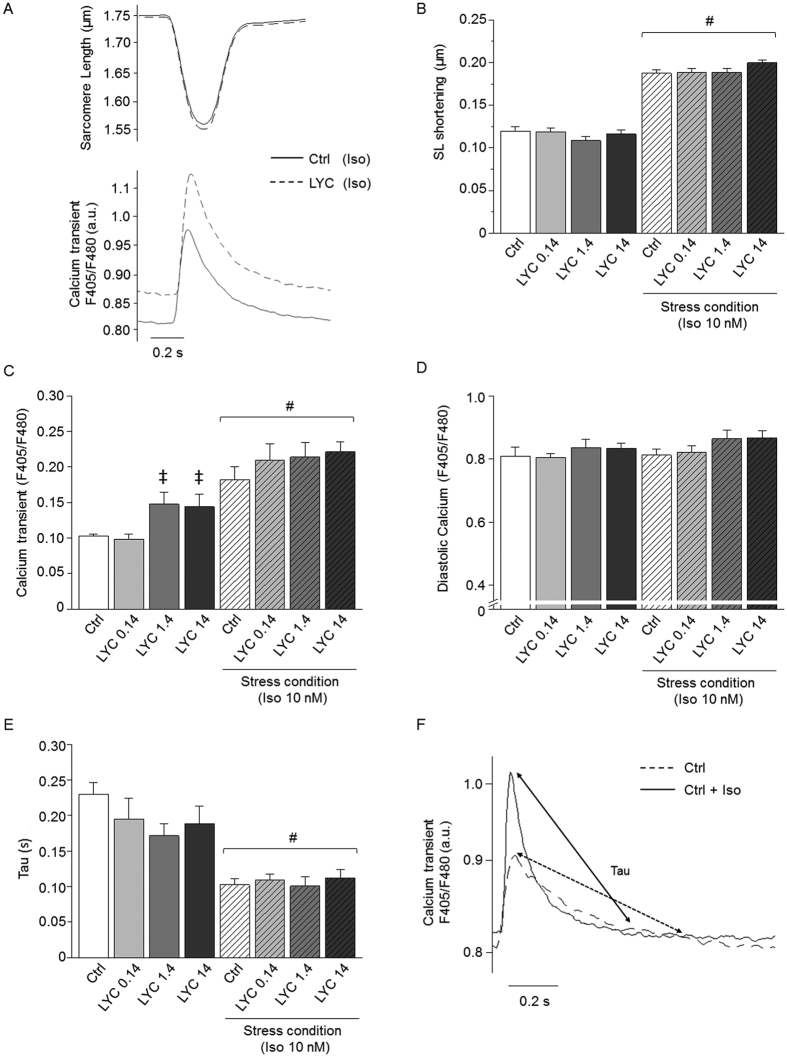
Acute exposure to LYC altered cellular Ca^2+^ handling in a dose-dependent manner in single cardiomyocytes from healthy mice. (**A**) Typical traces of the acute effect of LYC (14 nM) on sarcomere shortening (top panel) and Ca^2+^ transient (bottom panel) in presence of Iso (10 nM) B-F: Averaged acute effects of LYC at 0.14, 1.4 and 14 nM under basal and stress conditions (Iso 10 nM) on sarcomere shortening (**B**), Ca^2+^ transient amplitude (**C**), diastolic Ca^2+^ levels (**D**), and decay of Ca^2+^ transients. (**E**) Tau (time constant). Ca^2+^ is expressed as the ratio of fluorescence at 405 and 480 nm. (**F**) Typical traces of Ca^2+^ transient decay under control and Iso conditions. ^‡^p < 0.05, *vs*. Ctrl under the same conditions; ^#^p < 0.05, basal *vs*. stress condition; 10 cells per group.

**Figure 5:**
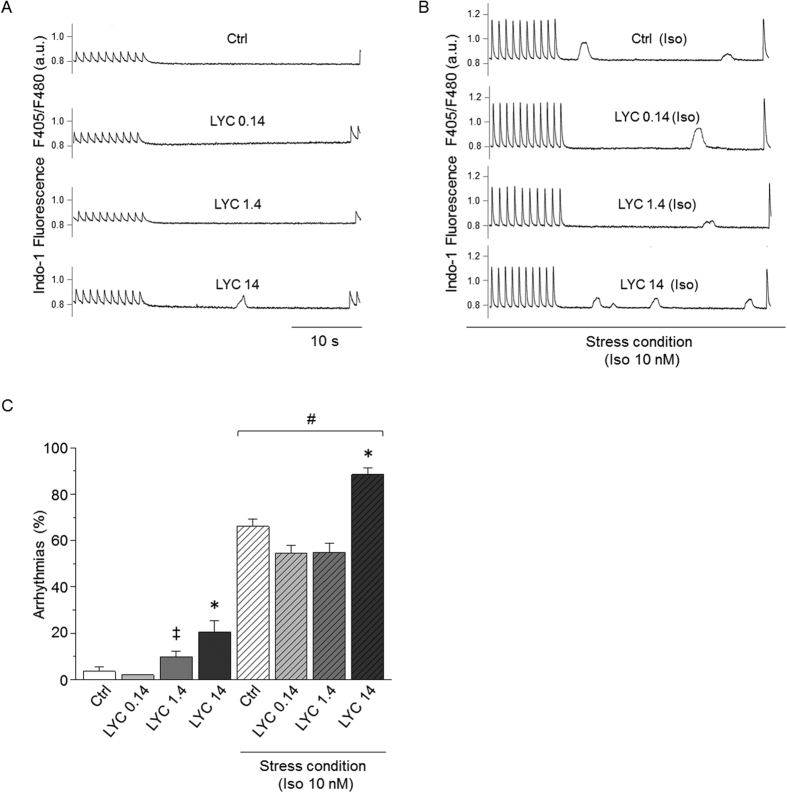
Acute exposure to LYC induced abnormal diastolic Ca^2+^ waves in a dose-dependent manner in cardiomyocytes from healthy mice. Representative traces of spontaneous Ca^2+^ waves during resting periods following trains of stimulation in single cardiomyocytes, under basal (**A**) and stress conditions (Iso 10 nM) (**B**). (**C**) Effect of increasing doses of LYC (0.14, 1.4 and 14 nM) on the occurrence of Ca^2+^ waves. Results are expressed as the percentage of cells exhibiting at least one Ca^2+^ wave; *p < 0.05, LYC *vs*. other groups under the same conditions; ^‡^p < 0.05, *vs*. Ctrl under the same conditions; ^#^p < 0.05, basal *vs*. stress condition; 20–30 cells per group.

We next investigated *in vitro* the acute cellular and molecular effects of LYC on Ca^2+^ handling in cardiomyocytes freshly isolated from healthy animals. We tested increasing concentrations of LYC (0.14 nM, 1.4 nM and 14 nM) on contractility and intracellular Ca^2+^ cycling. Under basal conditions, LYC increased the Ca^2+^ transient amplitude in a dose-dependent manner at 1.4 and 14 nM, although no significant effect was seen on sarcomere shortening (Fig. 4A–C) possibly reflecting a decreased sensitivity of contractile proteins to Ca^2+^. LYC had no significant effects on either the diastolic Ca^2+^ or the Ca^2+^ transient decay (Fig. 4D,E and F). However, strikingly, abnormal spontaneous Ca^2+^ waves still occurred during resting periods at the two highest LYC concentrations (Fig. 5A,C). Their occurrence was increased by a factor of 2 at 1.4 nM and a factor of 4 at 14 nM. As expected, the acute exposure of cardiomyocytes to Iso increased sarcomere shortening and Ca^2+^ transient amplitude (Fig. 4A–C) whilst accelerating Ca^2+^ transient decay in all experimental groups, due to the well-known enhanced pumping of Ca^2+^ via SERCA2a (Fig. 4E,F). During the Iso challenge, LYC did not promote substantial effects on sarcomere shortening, Ca^2+^ transient amplitude and diastolic Ca^2+^ (Fig. 4A–E). Although Iso promoted the occurrence of Ca^2+^ waves under all experimental conditions, this propensity was aggravated by LYC at 14 nM with nearly all cells exhibiting aberrant spontaneous events (Fig. 5B,C).

**Figure 6:**
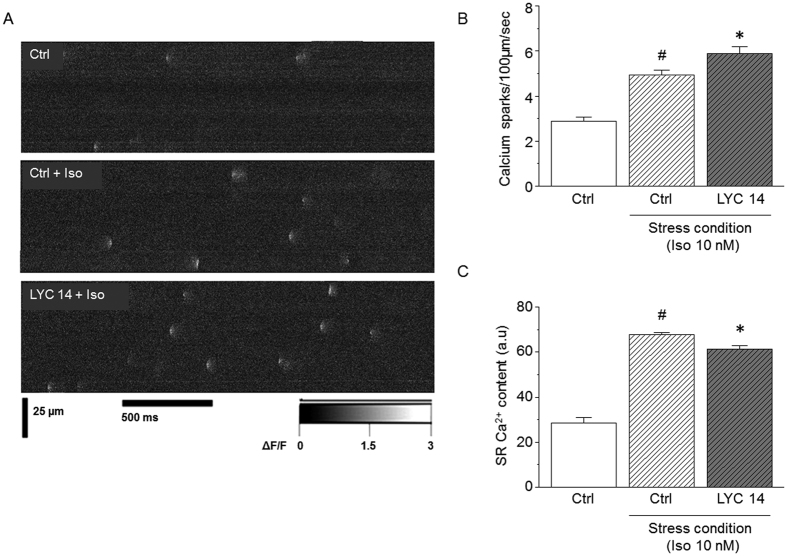
Acute exposure to LYC promoted spontaneous Ca^2+^ sparks. (**A**) Typical line-scan confocal images of Ca^2+^ sparks in Fluo-4-AM-loaded cardiomyocytes from mice treated with vehicle (Ctrl) or LYC (14 nM), in the presence or absence of Iso (10 nM). (**B**) Mean frequency of Ca^2+^ sparks measured under the different experimental conditions. (**C**) Averaged SR Ca^2+^ content in the different experimental conditions estimated from the caffeine-induced massive release of SR Ca^2+^ (expressed as the ratio of fluorescence at 405 and 480 nm); *p < 0.05, LYC 14 nM *vs*. Ctrl under the same condition; ^#^p < 0.05, basal *vs*. stress condition; 30–35 cells per group.

Spontaneous Ca^2+^ waves during diastolic periods frequently occur as a result of the abnormal opening of the ryanodine receptor 2 (RyR2)^15^,^16^,^17^,^18^. These microscopic Ca^2+^ events, referred to as Ca^2+^ sparks, can be assessed visually by means of confocal microscopy and the use of the fluorescent Ca^2+^ indicator Fluo-4 AM. We therefore evaluated the leakiness of RyR2 by measuring Ca^2+^ spark frequency. Since Iso had a major effect on the effect of LYC on the triggering of Ca^2+^ waves (Fig. 5B,C), we assessed the effect of 14 nM LYC on Ca^2+^ sparks in the presence of a maximally active concentration of Iso (Fig. 6). Our results showed that, as expected^19^, Iso increased the occurrence of Ca^2+^ sparks by a factor of ~2 (Fig. 6A,B). LYC further aggravated the occurrence of Ca^2+^ sparks. Since both Ca^2+^ transient amplitude and RyR2 activity depend, at least in part, on the Ca^2+^ content of the sarcoplasmic reticulum (SR), we estimated the SR Ca^2+^ content by triggering maximal RyR2-mediated Ca^2+^ release following rapid caffeine application (10 mM). The SR Ca^2+^ content was lowered by free LYC (Fig. 6C), in line with the promotion of SR Ca^2+^ leakage through RyR2. Altogether, our results reveal dose-dependent acute effects of LYC on Ca^2+^ handling both at the cellular level (Ca^2+^ transients, Ca^2+^ waves) and at the molecular level (Ca^2+^ sparks), which could be maintained following a β-adrenergic challenge suggesting additive mechanisms. In an additional set of experiments performed using the patch-clamp technique, we found that LYC had no acute effect on the cellular action potential (Supplemental data).

## Discussion

Sesquiterpenic lactones are molecules of natural origin which are emerging as promising source of bioactive prototype molecules with potential activity against *Trypanosoma cruzi*^20^,^21^. Treatment with LYC in PLA-PEG NC in acute and chronic phases of the infection has produced potent anti-trypanosomal efficacy in a mice model and may represent a valuable alternative to reference drugs benznidazole and nifurtimox, in particular in the case of *T. cruzi* strains resistant to these drugs^12^,^22^. However, on the basis of our previously reported results, a number of issues have arisen. Like most drugs, LYC has other biological activities, including antitumoral effects *in vitro*, as well as potential cytotoxicity^23^,^24^,^25^. Here, we show that repeated-dose administration of free LYC can potentially promote severe cardiotoxicity in mice, as evidenced by alterations of both global heart function and intracellular Ca^2+^ handling, with a critical effect on the RyR2 channel. However, a central finding of our study is that LYC encapsulated in biodegradable NC was safer with no adverse cardiac effects, indicating that this pharmaceutical formulation of LYC not only hold promise for curing CD^12^,^22^ but also in terms of cardiac safety.

Free LYC administered to C57BL/6 mice for 20 days (2.0 mg/kg/day) induced high mortality (50%), starting after 10 days of treatment, and severely altered cardiac function in the surviving animals. However, these mice had normal systolic function (i.e. preserved EF) but exhibited LV hypertrophy with increased wall thickness, reduced chamber dimension, and diastolic dysfunction (Fig. 1). The heart was able to eject a normal stroke volume but unable to accept a normal venous return, consistent with early concentric LV hypertrophy commonly seen in HF with preserved ejection fraction. This hypertrophic adaptation helps to maintain or enhance blood ejection but, at the same time, compromises chamber filling due to LV stiffness and, thereby, leads to a progressive deterioration of myocardial function until end-stage failure^26^,^27^,^28^. The ballooned apex of the heart was reminiscent of broken heart syndrome or drug-induced Tako-tsubo cardiomyopathy^13^,^14^.

Our findings at the cellular level support the view of an inotropic effect of LYC^29^. In mice surviving the 20-day LYC treatment, which were characterized by preserved ejection fraction with a lack of echocardiographic signs of decompensation, both the contractility and Ca^2+^ transient amplitude of cardiomyocytes were increased. Overall, our results are consistent with a compensatory state of the LV myocytes, described early after myocardial infarction in mice and accounted for changes in cellular excitation-contraction coupling, especially an increase of the Ca^2+^ transient amplitude^30^. However, these modifications were associated with abnormal macroscopic and microscopic diastolic Ca^2+^ events, namely Ca^2+^ waves and Ca^2+^ sparks respectively, in line with many studies on HF^15^,^31^,^32^.

LYC applied acutely in the nanomolar range (>1 nM) in isolated cardiomyocytes reproduced dose-dependently the effects on Ca^2+^ handling seen during *in vivo* repeated-dose treatment. In particular, LYC increased Ca^2+^ transient amplitude and promoted Ca^2+^ sparks reflecting RyR2-mediated diastolic Ca^2+^ leakiness responsible for the spontaneous firing of abnormal Ca^2+^ waves as found in inherited diseases related to RyR2 mutations, chronic diseases such as HF, or adverse drug effects^16^,^17^,^18^,^31^,^32^,^33^,^34^,^35^. The promotion of Ca^2+^ sparks/Ca^2+^ waves by LYC remained during β-adrenergic receptor stimulation (Fig. 4), i.e. over and above the effect of the latter^19^, suggesting that LYC induces RyR2 leakage through increased sensitivity for opening via an additive mechanism. The molecular/cellular cause of the increased Ca^2+^ transient amplitude was unclear and puzzling. A thapsigargin-like inhibition of calcium ATPase (SERCA2a) pumps or SERCA orthologues (e.g. PfATP6 in *Plasmodium falciparum*) by sesquiterpenic lactones has been reported^36^,^37^. However, we had no experimental evidence for such an acute inhibitory effect of LYC on SERCA2a. In particular, LYC did not slow the fast Ca^2+^ transient decay, or decreased the Ca^2+^ transient nor increased dramatically diastolic Ca^2+^ as shown with thapsigargin^38^. Of note, normal SERCA2a activity may compensate for Ca^2+^ leakage through RyR2 by an efficient Ca^2+^ pumping in the SR to attenuate diastolic Ca^2+^ overload.

The acute effects of free-LYC involved increased Ca^2+^ transient amplitude, but this was however associated with a disruption of normal Ca^2+^ cycling fostering abnormal Ca^2+^ sparks and Ca^2+^ waves (promoted by Ca^2+^ sparks) in line with RyR2 being a primary target of LYC effects. Interestingly, the trypanocidal activity of sesquiterpene lactones was reported to inactivate trypanosome defense against oxidative stresses^39^. Sesquiterpene lactones can act as oxidative stress inducers^23^, which may account for the LYC damages observed herein. For example, LYC, deoxyelephantopin^40^ and parthenolide^41^ have in common α-methylene-γ-lactones groups that react by the Michael-type addition with mercaptyl groups of cysteine residues in the free intracellular glutathione, leading to reduced activity and altered redox balance^23^. Therefore, LYC may promote similar oxidative stress–mediated cell damage through Reactive Oxygen Species (ROS) in cardiomyocytes.

Moderate oxidative stress causes pathological change in RyR2 conformation and increases both Ca^2+^ sparks frequency and Ca^2+^ waves, as seen in heart failure^42^, and in line with our results in this study. Redox-sensitive alterations of Na^+^ and Ca^2+^ handling proteins as well as modifications of key regulating kinases of these proteins have critical physiological roles and high pathological relevance^43^. Interestingly, Na^+^ overload due to ROS-dependent activation of sustained Na^+^ currents may participate to increase SR Ca^2+^ load^32^,^44^ thereby explaining the concomitant increase of the Ca^2+^ transient and the RyR2 leakage induced by LYC. These mechanisms are highly pathophysiologic^43^. The Ca^2+^ handling alterations induced by LYC may contribute to the pathogenesis and progression of the LYC-induced cardiopathy described in this study. Leaky RyR2 channels are known to activate pathological hypertrophic pathways responsible for cardiac remodelling and deterioration^31^,^45^,^46^,^47^. Direct oxidative modifications of myofilament proteins, myofilament protein phosphorylation by ROS-activated kinases, or myofilament protein cleavage by ROS-activated proteases can also interfere with the transduction of calcium-dependent contractile responses (see review, ref. 48). Cardiac contraction typically is reduced following treatment with oxidizing agents which could explain the apparent discrepancy of the acute effect of LYC on the Ca^2+^ transient (positive inotropism) and contraction (no effect) in our study. Interestingly, polyester-based NC obtained from PLA-PEG polymer produce no oxidative stress even at high concentrations^49^,^50^ and could protect the heart of mice against excessive oxidative stress induced by LYC, particularly related to NC ability to control the LYC release in plasma. This hypothesis warrants further investigations.

We have previously shown that LYC encapsulated in long-circulating PLA-PEG NC improves dramatically the anti-trypanosomal properties of LYC by *iv* route, increasing infected-mice survival (100% LYC-NC *versus* 0% free-LYC) at the chronic phase^22^. Furthermore, only LYC in PLA-PEG NC formulation provides mice cure by intravenous route (50% LYC-NC *versus* 0% free-LYC)^12^,^22^. The efficacy of LYC was shown to be improved by encapsulation in the biodegradable sterically stabilized polymeric PLA-PEG NC formulations administered intravenously in acute and chronic phases of the disease^12^,^22^. Free LYC is able to reduce parasitemia and increase survival in infected animals, however no animal cure was observed in both stages of the infection. Furthermore, after intravenous injection of a single dose of 12.6 mg/kg, the LYC plasma clearance is 39 and 2.3 mL/min in mice for free LYC and LYC-PLA-PEG NC, respectively (paper submitted to *Scientific Reports*). This shows clearly that nanoencapsulation reduces the LYC clearance from mice plasma. Considering these results, our next step was to assess the possible cardiotoxicity of this new drug candidate and, obviously, also of LYC associated to NC formulation. A key finding of our study was that the severe adverse effects of LYC with regard to mortality and cardiac pathogenesis were prevented by encapsulation of the LYC. Not only were the effects of empty NC neutral, enabling us to distinguish between the potential toxicity of LYC and that of the NC, but the encapsulated LYC had also no harmful effects on the parameters evaluated, including myocardial function and morphology, cardiomyocyte contraction, Ca^2+^ transients and the occurrence of Ca^2+^ sparks and related Ca^2+^ waves, unlike the effects of free LYC, even under challenging conditions such as β-adrenergic stress. Our pharmaceutical formulation of LYC encapsulated in PLA-PEG NC therefore appears to be a promising step forward in providing a potential therapeutic option to treat CD with minimal cardiotoxicity. How can this differential effect of encapsulated LYC, i.e. improved anti-trypanosomal activity on the one hand and cardiac protection on the other, be explained? Long-circulating PLA-PEG NC improved LYC efficacy^12^,^22^, increased 26-times body exposure to the LYC and controlled its release even in mice plasma (Branquinho *et al*., paper submitted in *Scientific Reports*). Previously reported data showed that encapsulation has a profound influence in the amount of LYC available in the plasma to interact with target sites and non-target sites promoting side effects. As a significant part of LYC circulates in plasma inside NC, free drug toxic effects may be diminished by reducing interactions with the heart. Oppositely, the LYC-NC with their reduced sizes (<200 nm) can extravasate and accumulate more in tissues compromised by inflammation process, which have leaky endothelium^51^. Thus, differences in the efficacy can be explained by the differences in LYC biodistribution, accumulation in the infectious/inflammatory sites and also by controlled release of LYC inside blood compartment. Furthermore, our previous results show that NC protects LYC from degradation in mice plasma extending the parent molecule duration of the pharmacological effects. The NC long-circulating properties and the LYC controlled release (80% within 6 h) provided by NC may be more effective than massive *bolus* injections of free LYC once a day to cure infection^22^. Minimal cardiac effects are then expected at these low released doses of LYC from NC inside blood. The reduction of cardiotoxicity may also reflect the ability of nanocarriers to modify the distribution of the entrapped drug in the body, as shown for halofantrine^52^, with a lower fraction of free drug available for interaction with cardiac tissue, in line with the lack of an effect of LYC in solution at subnanomolar concentrations on cardiomyocytes as shown here (Figs 4 and 5; 0.14 nM *vs.* 1.4 nM and 14 nM). This may be particularly interesting for repeated-dose oral treatment during chronic infection. Indeed, no obvious adverse effects and no death were observed in *T. cruzi* infected-mice treated at chronic phase with LYC-PLA-PEG NC^22^, in line with the study herein of *iv* administration on healthy animals. Oppositely, 50% and 60% mortality occurred in healthy animals treated with free-LYC and in animals *T. cruzi*-infected (chronic phase)^22^, respectively. High mortality in infected animals is expected, because infection is lethal (50% mortality in infected non–treated groups)^22^. Mice treated *iv* during 20 days at the acute phase of infection with free-LYC (n = 16), 19% died during treatment and 30% after the end of the treatment^12^. Although the cardiac complications contributing to death could not be identified in this latter study, general toxicity may be also involved and will be the subject of further investigations. Of note, C57BL/6 mice are more susceptible to oxidative stress than Swiss mice^53^, which may potentially lead to differences in cardiotoxicity between the two strains (Swiss *vs.* C57BL/6). For example, these mice present lower levels of glutathione-related antioxidant defenses in the hippocampus and prefrontal cortex^54^.

There is still a long way to go before considering the application of the therapeutic principles described here to human health. The control of adverse effects may be species-dependent and even variable. An assessment of LYC cardiotoxicity in mice with CD is also clearly warranted, although performing all the investigations reported here in infected animals is a technical challenge to provide the adequate safety conditions for experimenters to run all the experimental approaches used in our study. It will also be important to explore whether LYC is toxic to other organs/functions, and whether these different risks can likewise be prevented by nanoencapsulation.

In summary, we have tested for the first time in an experimental model potential adverse effects of LYC at the cardiac level. Although we evidenced a cardiotoxicity during repeated-dose treatment regimen with LYC administered in its free form, we also unravelled a remarkable protection against this cardiotoxicity when LYC was encapsulated in polymeric NC. Importantly, this concept may open new prospects for treatment of the chronic phase of CD, as well as the treatment of other parasitic diseases, and antitumoral therapy with LYC. This study is thus promising but warrants the further evaluation of NC-encapsulated LYC prior to its development for clinical use against different strains of *T. cruzi* as well as for its antitumoral properties. Overall, we highlight an important contribution of nanotechnology not only in improving the efficacy of a drug (here LYC against *T. cruzi* infection), but also its potential in protecting against adverse effects.

## Methods

### Preparation of free LYC solution and LYC-loaded nanocapsules

LYC loaded in sterically stabilized NC (LYC-NC) with a PEG corona at the surface was prepared via the interfacial deposition of the preformed polymer followed by solvent displacement, using a monomethoxy-polyethylene glycol-*block*-poly(lactide) polymer as described^11^,^12^ (part I submitted to Sci. Reports). Blank NC were prepared as controls using the same protocol as for LYC-loaded NC, but by omitting LYC. The mean hydrodynamic diameter and polydispersity index of the NC population was determined by dynamic light scattering (Zetasizer Nano ZS, Malvern Instruments, UK) as described^55^,^56^. LYC solution (2 mg/mL) filtered at 0.22 μm, suitable for intravenous administration, was used^12^. Briefly, LYC was dissolved in a N,N-dimethylacetamide: polyethylene glycol 300 (DMA:PEG) 4:6 v/v mixture and further diluted in isotonic glucose solution to attain its final concentration. The control group received excipients of the intravenous solution^11^,^12^. All nanocapsule formulations were filtered through 0.45 μm-pore-size sterile filters immediately after preparation and before intravenous administration. More than 90% of encapsulated LYC can be found in the oily core of NC, and diffusion through membranes (dialysis) occurs slowly with approximately 60% of the drug being released after 24 h^11^.

### Animals and cells isolation

All procedures conformed to European Parliament Directive 2010/63/EU, the 22 September 2010 Council on animal protection, and NIH Guidelines for the Care and Use of Laboratory Animals. The project was approved by the French *Ministère de la Recherche et de l’Enseignement Supérieur* (N° 02571.01). Seven-week-old male C57BL/6 mice (Janvier, Le Genest-Saint-Isle, France) were used. To study the repeated-dose effects of treatments, animals received daily intravenous injections of free LYC solution (2.0 mg/kg/day; 8 mice), LYC loaded in biodegradable polymeric NC (LYC-NC; 2.0 mg/kg/day, 10 mice), blank-NC (10 mice) and vehicle (Ctrl group; 10 mice) for 20 consecutive days before echocardiographic evaluation at day 19 and sacrifice at day 20, according the classical *in vivo* protocol of Chagas disease screening of efficacy. This is also a 20^th^ day repeated-dose toxicological protocol for studies of active substances against *T. cruzi* in mice model^57^. The acute effects of LYC were studied at 0.14 nM, 1.4 nM and 14 nM on single cardiomyocytes obtained from Ctrl animals. In each mouse, the heart was rapidly excised after euthanasia (cervical dislocation) and submitted to enzymatic action (liberase) using a Langendorff perfusion system in order to disperse single rod-shaped left ventricular (LV) myocytes^16^,^17^,^58^. Only cardiomyocytes with clear edges were used within 1–6 h of isolation. Cardiomyocytes with eventual spontaneous contractions were excluded.

### Echocardiography

Transthoracic echocardiography was performed with the high-resolution imaging system Vevo2100 (VisualSonics Fujifilm inc.) equipped with a 40 MHz probe on the platform *Small animal imaging platform* (certified norme ISO 9001: 2008). Mice were anesthetized with isoflurane (1–2%, in 100% oxygen). Body temperature and heart beat were monitored throughout the procedure and maintained at physiological levels (36 ± 1 °C and 436 ± 36 bpm, respectively). Wall thickness and left ventricular diameter were measured at the level of the papillary muscles in a parasternal long-axis two-dimensional view by M-mode, allowing the calculation of the EF by the Teicholz method and the relative wall thickness (RWT)^28^. Mitral inflow was recorded by pulsed-wave Doppler in an apical four-chamber view by placing sample at the tip of the mitral valves level. The velocities of peak early (E) and late atrial contraction (A) mitral inflow waves were measured, and the E/A ratio was calculated as an index of LV diastolic function. All measurements were performed in triplicate. See more detailed methods in the Supplementary Material.

### Single-cell contractility and real-time Ca^2+^ imaging

Contractility and intracellular free Ca^2+^ were measured in single LV myocytes field-stimulated at 1.0 Hz with 1-ms current pulses to assess cell shortening and Ca^2+^ transients, respectively, as described^16^,^17^,^38^,^58^. Briefly, cardiomyocytes were bathed in a physiological solution (in mM: 117 NaCl, 5.7 KCl, 4.4 NaHCO_3_, 1.5 KH_2_PO_4_, 1.7 MgCl_2_, 21 HEPES, 1.8 CaCl_2_ and 11 glucose). Sarcomere length (SL) and Ca^2+^-sensitive fluorescence (405 and 480 nm) were simultaneously recorded using an IonOptix system (Milton, MA, USA) and a Zeiss microscope (40X oil-immersion objective, 0.36 μm/pixel). To monitor intracellular Ca^2+^, cardiomyocytes were loaded with the dual-emission ratiometric Ca^2+^ indicator Indo-1AM (2 μM, Invitrogen, Grand Island, NY, USA), which emits at 405 nm and 480 nm concurrently. They were simultaneously illuminated at 365 nm using a xenon arc lamp. Cytosolic Ca^2+^ concentration was determined by the ratio of 405 nm/480 nm fluorescence (a.u.: arbitrary units). Cell shortening, diastolic Ca^2+^ levels and Ca^2+^ transient amplitude were measured. Sarcoplasmic reticulum (SR) Ca^2+^ content was estimated by massive RyR2 channel opening by means of caffeine (10 mM). To assess diastolic events, cells were stimulated by trains of stimulations during 30 s periods (1.0 Hz) followed by a 30 s rest period. Cells developing spontaneous Ca^2+^ waves and contractions during the diastolic period were identified. Cells were also challenged under conditions of stress triggered by exposure to the β1-adrenergic agonist isoproterenol (Iso, 10 nM) for 10 minutes before experiments. Data were analyzed using Ionwizard Software.

Ca^2+^ sparks were recorded in quiescent cells loaded with the Ca^2+^ indicator Fluo-4 AM (4 μM, Molecular Probes, Paris, France) and a confocal microscope in line-scan mode (1.5 ms/line, 512 pixels × 3000 lines, LSM510 Zeiss confocal microscope; 63X water-immersion objective, N.A.: 1.2) at 25 °C. The dye was excited at 488 nm and the fluorescence emitted collected through a 505-nm long-pass filter. Myocytes were field-stimulated at 1 Hz with 1-ms current pulses delivered via two platinum electrodes, one on each side of the perfusion chamber. During the rest period that followed stimulation, myocytes were repetitively scanned along their entire length at 1.5-ms intervals, for a maximum of 6 s^17^,^58^,^59^. The intensity of the laser was reduced to 5% of maximum to decrease cell damage and dye bleaching. Line-scan diagrams were constructed by stacking emission lines, corresponding to excitation scans, in temporal order. An average of the Ca^2+^ sparks was determined by the intensity of each sequential scan line and plotting the mean intensity as a function of time. The SparkMaster plug-in for ImageJ software was used to detect and analyze Ca^2+^ sparks.

### Statistical analysis

All data are expressed as means ± SEM. Statistical analyses were performed using GraphPad Prism^®^ (Prism 5 for Mac OS X). For comparison of Survival curves, we used Log-rank (Mantel-Cox) test. For cellular experiments and studies of Ca^2+^ sparks, 10–35 cells per group were used for each experimental condition. For multiple comparisons, one-way ANOVA was used, followed by a parametric t-test with Fisher’s correction. For comparison of two conditions in the same group (baseline *vs*. iso) a paired Student *t*-test was used. A p-value of 0.05 or less was taken to indicate statistical significance.

## Additional Information

**How to cite this article:** Branquinho, R. T. *et al*. Biodegradable Polymeric Nanocapsules Prevent Cardiotoxicity Of Anti-Trypanosomal Lychnopholide. *Sci. Rep.* **7**, 44998; doi: 10.1038/srep44998 (2017).

**Publisher's note:** Springer Nature remains neutral with regard to jurisdictional claims in published maps and institutional affiliations.

## Supplementary Material

Supplementary Data

## References

[b1] Nunes M. C. P. . Chagas disease: an overview of clinical and epidemiological aspects. J. Am. Coll. Cardiol. 62, 767–776 (2013). 23770163 10.1016/j.jacc.2013.05.046

[b2] Prata A. Clinical and epidemiological aspects of Chagas disease. Lancet Infect Dis 1, 92–100 (2001). 11871482 10.1016/S1473-3099(01)00065-2

[b3] Rassi A., Rassi A. & Marin-Neto J. A. Chagas disease. Lancet 375, 1388–1402 (2010). 20399979 10.1016/S0140-6736(10)60061-X

[b4] Ribeiro A. L., Nunes M. P., Teixeira M. M. & Rocha M. O. C. Diagnosis and management of Chagas disease and cardiomyopathy. Nat Rev Cardiol 9, 576–589 (2012). 22847166 10.1038/nrcardio.2012.109

[b5] Biolo A., Ribeiro A. L. & Clausell N. Chagas cardiomyopathy–where do we stand after a hundred years? Prog Cardiovasc Dis 52, 300–316 (2010). 20109600 10.1016/j.pcad.2009.11.008

[b6] Bern C. Chagas’ Disease. N. Engl. J. Med. 373, 1882 (2015). 10.1056/NEJMc1510996 26535522

[b7] Molina I. . Randomized trial of posaconazole and benznidazole for chronic Chagas’ disease. N. Engl. J. Med. 370, 1899–1908 (2014). 24827034 10.1056/NEJMoa1313122

[b8] Rodriques Coura J. & de Castro S. L. A critical review on Chagas disease chemotherapy. Mem. Inst. Oswaldo Cruz 97, 3–24 (2002). 11992141 10.1590/s0074-02762002000100001

[b9] Viotti R. . Side effects of benznidazole as treatment in chronic Chagas disease: fears and realities. Expert Rev Anti Infect Ther 7, 157–163 (2009). 19254164 10.1586/14787210.7.2.157

[b10] Bustamante J. M. & Tarleton R. L. Potential new clinical therapies for Chagas disease. Expert Rev Clin Pharmacol 7, 317–325 (2014). 24716790 10.1586/17512433.2014.909282

[b11] Branquinho R. T. . HPLC-DAD and UV-spectrophotometry for the determination of lychnopholide in nanocapsule dosage form: validation and application to release kinetic study. J Chromatogr Sci 52, 19–26 (2014). 23247030 10.1093/chromsci/bms199

[b12] Branquinho R. T. . Sesquiterpene lactone in nanostructured parenteral dosage form is efficacious in experimental Chagas disease. Antimicrob. Agents Chemother. 58, 2067–2075 (2014). 24449777 10.1128/AAC.00617-13 PMC4023798

[b13] Naser N. . The role of echocardiography in diagnosis and follow up of patients with takotsubo cardiomyopathy or acute ballooning syndrome. Med Arh 65, 287–290 (2011). 22073853 10.5455/medarh.2011.65.287-290

[b14] Amariles P. & Cifuentes L. Drugs as Possible Triggers of Takotsubo Cardiomyopathy: a Comprehensive Literature Search - Update 2015. *Curr Clin Pharmacol* (2016). 10.2174/1574884711666160405105841 27049039

[b15] Cheng H. & Lederer W. J. Calcium sparks. Physiol. Rev. 88, 1491–1545 (2008). 18923188 10.1152/physrev.00030.2007

[b16] Fauconnier J. . Leaky RyR2 trigger ventricular arrhythmias in Duchenne muscular dystrophy. Proc. Natl. Acad. Sci. USA 107, 1559–1564 (2010). 20080623 10.1073/pnas.0908540107 PMC2824377

[b17] Thireau J. . Β-adrenergic blockade combined with subcutaneous B-type natriuretic peptide: a promising approach to reduce ventricular arrhythmia in heart failure? Heart 100, 833–841 (2014). 24667281 10.1136/heartjnl-2013-305167

[b18] Fernández-Velasco M. . Increased Ca2+ sensitivity of the ryanodine receptor mutant RyR2R4496C underlies catecholaminergic polymorphic ventricular tachycardia. Circ. Res. 104, 201–209, 12p following 209 (2009). 19096022 10.1161/CIRCRESAHA.108.177493 PMC2796688

[b19] Santiago D. J., Ríos E. & Shannon T. R. Isoproterenol increases the fraction of spark-dependent RyR-mediated leak in ventricular myocytes. Biophys. J. 104, 976–985 (2013). 23473480 10.1016/j.bpj.2013.01.026 PMC3591256

[b20] Fabian L. . In silico study of structural and geometrical requirements of natural sesquiterpene lactones with trypanocidal activity. Mini Rev Med Chem 13, 1407–1414 (2013). 23815577 10.2174/13895575113139990066

[b21] Jimenez V. . Natural sesquiterpene lactones induce programmed cell death in Trypanosoma cruzi: a new therapeutic target? Phytomedicine 21, 1411–1418 (2014). 25022207 10.1016/j.phymed.2014.06.005

[b22] de Mello C. G. C. . Efficacy of lychnopholide polymeric nanocapsules after oral and intravenous administration in murine experimental Chagas disease. Antimicrob. Agents Chemother, doi: 10.1128/AAC.00178-16 (2016). PMC4997880 27324760

[b23] Gach K., Długosz A. & Janecka A. The role of oxidative stress in anticancer activity of sesquiterpene lactones. Naunyn Schmiedebergs Arch. Pharmacol. 388, 477–486 (2015). 25656627 10.1007/s00210-015-1096-3

[b24] Salla M., Fakhoury I., Saliba N., Darwiche N. & Gali-Muhtasib H. Synergistic anticancer activities of the plant-derived sesquiterpene lactones salograviolide A and iso-seco-tanapartholide. J Nat Med 67, 468–479 (2013). 22976170 10.1007/s11418-012-0703-6

[b25] Saúde-Guimarães D. A., Raslan D. S. & Oliveira A. B. *In vitro* antitumor activity of sesquiterpene lactones from Lychnophora trichocarpha. Rev Bras Plantas Med 201416, 275–282 (2014).

[b26] Katz A. M. & Rolett E. L. Heart failure: when form fails to follow function. Eur. Heart J. 37, 449–454 (2016). 26497163 10.1093/eurheartj/ehv548

[b27] Braunwald E. Research Advances in Heart Failure A Compendium. Circ Res 113, 633–645 (2013). 23888056 10.1161/CIRCRESAHA.113.302254

[b28] Milani R. V. . Left ventricular geometry and survival in patients with normal left ventricular ejection fraction. Am. J. Cardiol. 97, 959–963 (2006). 16563894 10.1016/j.amjcard.2005.10.030

[b29] Hasenfuss G. & Pieske B. Calcium cycling in congestive heart failure. J. Mol. Cell. Cardiol. 34, 951–969 (2002). 12234765 10.1006/jmcc.2002.2037

[b30] Mørk H. K. . Increased cardiomyocyte function and Ca2+ transients in mice during early congestive heart failure. J. Mol. Cell. Cardiol. 43, 177–186 (2007). 17574269 10.1016/j.yjmcc.2007.05.004

[b31] Kushnir A. & Marks A. R. The ryanodine receptor in cardiac physiology and disease. Adv. Pharmacol. 59, 1–30 (2010). 20933197 10.1016/S1054-3589(10)59001-X PMC3023997

[b32] Thireau J., Pasquié J.-L., Martel E., Le Guennec J.-Y. & Richard S. New drugs vs. old concepts: a fresh look at antiarrhythmics. Pharmacol. Ther. 132, 125–145 (2011). 21420430 10.1016/j.pharmthera.2011.03.003

[b33] Priori S. G. & Chen S. R. W. Inherited dysfunction of sarcoplasmic reticulum Ca2+ handling and arrhythmogenesis. Circ. Res. 108, 871–883 (2011). 21454795 10.1161/CIRCRESAHA.110.226845 PMC3085083

[b34] Kashimura T. . In the RyR2(R4496C) mouse model of CPVT, β-adrenergic stimulation induces Ca waves by increasing SR Ca content and not by decreasing the threshold for Ca waves. Circ. Res. 107, 1483–1489 (2010). 20966392 10.1161/CIRCRESAHA.110.227744

[b35] Fauconnier J., Pasquié J.-L., Bideaux P., Lacampagne A. & Richard S. Cardiomyocytes hypertrophic status after myocardial infarction determines distinct types of arrhythmia: role of the ryanodine receptor. Prog. Biophys. Mol. Biol. 103, 71–80 (2010). 20109482 10.1016/j.pbiomolbio.2010.01.002

[b36] Eckstein-Ludwig U. . Artemisinins target the SERCA of Plasmodium falciparum. Nature 424, 957–961 (2003). 12931192 10.1038/nature01813

[b37] Krishna S., Pulcini S., Moore C. M., Teo B. H.-Y. & Staines H. M. Pumped up: reflections on PfATP6 as the target for artemisinins. Trends Pharmacol. Sci. 35, 4–11 (2014). 24268763 10.1016/j.tips.2013.10.007

[b38] Thireau J. . ACE Inhibitor Delapril Prevents Ca(2+)-Dependent Blunting of IK1 and Ventricular Arrhythmia in Ischemic Heart Disease. Curr. Mol. Med. 15, 642–651 (2015). 26321755 10.2174/1566524015666150831131459

[b39] Saeidnia S., Gohari A. R. & Haddadi A. Biogenic trypanocidal sesquiterpenes: lead compounds to design future trypanocidal drugs - a mini review. Daru 21, 35 (2013). 23676125 10.1186/2008-2231-21-35 PMC3663703

[b40] Mehmood T. . Deoxyelephantopin induces apoptosis in HepG2 cells via oxidative stress, NF-κB inhibition and mitochondrial dysfunction. Biofactors, doi: 10.1002/biof.1324 (2016). 27628030

[b41] Tsai T.-Y. . Parthenolide-Induced Cytotoxicity in H9c2 Cardiomyoblasts Involves Oxidative Stress. Acta Cardiol Sin 31, 33–41 (2015). 27122844 10.6515/ACS20140422B PMC4804911

[b42] Oda T. . Oxidation of ryanodine receptor (RyR) and calmodulin enhance Ca release and pathologically alter, RyR structure and calmodulin affinity. J. Mol. Cell. Cardiol. 85, 240–248 (2015). 26092277 10.1016/j.yjmcc.2015.06.009 PMC4530019

[b43] Sag C. M., Wagner S. & Maier L. S. Role of oxidants on calcium and sodium movement in healthy and diseased cardiac myocytes. Free Radic. Biol. Med. 63, 338–349 (2013). 23732518 10.1016/j.freeradbiomed.2013.05.035

[b44] Yang Z. . Epac2-Rap1 signaling regulates reactive oxygen species production and susceptibility to cardiac arrhythmias. Antioxid. Redox Signal, doi: 10.1089/ars.2015.6485 (2016). PMC5510674 27649969

[b45] Lehnart S. E. . Phosphodiesterase 4D deficiency in the ryanodine-receptor complex promotes heart failure and arrhythmias. Cell 123, 25–35 (2005). 16213210 10.1016/j.cell.2005.07.030 PMC2901878

[b46] Marx S. O. & Marks A. R. Dysfunctional ryanodine receptors in the heart: new insights into complex cardiovascular diseases. J. Mol. Cell. Cardiol. 58, 225–231 (2013). 23507255 10.1016/j.yjmcc.2013.03.005 PMC4042628

[b47] Heineke J. & Molkentin J. D. Regulation of cardiac hypertrophy by intracellular signalling pathways. Nat. Rev. Mol. Cell Biol. 7, 589–600 (2006). 16936699 10.1038/nrm1983

[b48] Steinberg S. F. Oxidative stress and sarcomeric proteins. Circ. Res. 112, 393–405 (2013). 23329794 10.1161/CIRCRESAHA.111.300496 PMC3595003

[b49] Sari E. . ICAM-1 targeted catalase encapsulated PLGA-b-PEG nanoparticles against vascular oxidative stress. J Microencapsul 32, 687–698 (2015). 26471402 10.3109/02652048.2015.1073384

[b50] Venkatpurwar V. P. . Drug- not carrier-dependent haematological and biochemical changes in a repeated dose study of cyclosporine encapsulated polyester nano- and micro-particles: size does not matter. Toxicology 330, 9–18 (2015). 25637670 10.1016/j.tox.2015.01.017

[b51] Pereira M. A. . Biodistribution study and identification of inflammatory sites using nanocapsules labeled with (99m)Tc-HMPAO. Nucl Med Commun 30, 749–755 (2009). 19593235 10.1097/MNM.0b013e32832f2b59

[b52] Leite E. A. . Cardiotoxicity reduction induced by halofantrine entrapped in nanocapsule devices. Life Sci. 80, 1327–1334 (2007). 17303179 10.1016/j.lfs.2006.12.019

[b53] Rueff-Barroso C. R. . Organ-related cigarette smoke-induced oxidative stress is strain-dependent. Med. Sci. Monit. 16, BR218–226 (2010). 20581770

[b54] Prediger R. D. S. . Differential susceptibility following beta-amyloid peptide-(1–40) administration in C57BL/6 and Swiss albino mice: Evidence for a dissociation between cognitive deficits and the glutathione system response. Behav. Brain Res. 177, 205–213 (2007). 17194489 10.1016/j.bbr.2006.11.032

[b55] Garcia G. M. . Improved nonclinical pharmacokinetics and biodistribution of a new PPAR pan-agonist and COX inhibitor in nanocapsule formulation. J Control Release 209, 207–218 (2015). 25931305 10.1016/j.jconrel.2015.04.033

[b56] de Paula C. S. . Chloroaluminium phthalocyanine polymeric nanoparticles as photosensitisers: photophysical and physicochemical characterisation, release and phototoxicity *in vitro*. Eur J Pharm Sci 49, 371–381 (2013). 23542495 10.1016/j.ejps.2013.03.011

[b57] Romanha A. J. . *In vitro* and *in vivo* experimental models for drug screening and development for Chagas disease. Mem. Inst. Oswaldo Cruz 105, 233–238 (2010). 20428688 10.1590/s0074-02762010000200022

[b58] Roy J. . Non-enzymatic lipid mediators, neuroprostanes, exert the anti-arrhythmic properties of docosahexaenoic acid. Free Radic. Biol. Med, doi: 10.1016/j.freeradbiomed.2015.04.014 (2015). 25911196

[b59] Fauconnier J. . Ryanodine receptor leak mediated by caspase-8 activation leads to left ventricular injury after myocardial ischemia-reperfusion. Proc. Natl. Acad. Sci. USA 108, 13258–13263 (2011). 21788490 10.1073/pnas.1100286108 PMC3156220

