# Supplementary material for: Biodegradable Polymeric Nanocapsules Prevent Cardiotoxicity of Anti-Trypanosomal Lychnopholide
Source: Sci Rep. 2017 Mar 28;7:44998. doi: 10.1038/srep44998 (PMC5368638; doi:10.1038/srep44998)
Supplement: Supplementary Data [file srep44998-s1.doc]

**BIODEGRADABLE POLYMERIC NANOCAPSULES PREVENT**

**CARDIOTOXICITY OF ANTI-TRYPANOSOMAL LYCHNOPHOLIDE**

Cardiac effects of new drug to treat Chagas disease

Renata Tupinambá Branquinho^1,2^,*, Jérôme Roy^2^,*, Charlotte Farah^2^,*, Giani Martins Garcia^1,2^, Franck Aimond^2^, Jean-Yves Le Guennec^2^, Dênia Antunes Saude-Guimarães^1^; Andrea Grabe-Guimaraes^1^, Vanessa Carla Furtado Mosqueira^1,§^; Marta de Lana^1,§^, Sylvain Richard^2,§^

*: equal contributions.

§: equal contributions.

1: Pharmaceutical Sciences Post-graduation Program (CiPharma), Escola de Farmácia, Universidade Federal de Ouro Preto, Minas Gerais, Brazil.

2: PHYMEDEXP, Inserm U1046, CNRS UMR 9214, Université de Montpellier – Montpellier, France.

Corresponding author:

Sylvain Richard

Inserm 1046 - UMR CNRS 9214 PhyMedExp

CHU Arnaud de Villeneuve, Bâtiment Crastes de Paulet

371 avenue du doyen Gaston Giraud

34295 MONTPELLIER Cedex 5, (FRANCE)

Phone: +33 467 415 241

Fax: +33 467 415 242

Email: [sylvain.richard@inserm.fr](mailto:sylvain.richard@inserm.fr)

**Supplementary Table 1:**

Heart morphology and function assessed by transthoracic echocardiography after 20 days of treatment.

**HR**: heart rate; **IVSd**: Inter-ventricular septum thickness in diastole; **IVSs**: Inter-ventricular septum thickness in systole; **LVIDd**: left ventricular internal diameter in diastole; **LVIDs**: left ventricular internal diameter in systole; **PWTd**: posterior wall thickness in diastole; **PWTs**: posterior wall thickness in systole; **EF**: ejection fraction measured by the Teicholz method; **FS**: fractional shortening measured by the Teicholz method; **EF B-mode**: ejection fraction measured by the Simpson method in B-mode; **RWT**: relative wall thickness, calculated as RWT = (IVSd + IVSs) / LVIDd; **Ao VTI**: aortic flow velocity-time integral; **E wave**: peak velocity of early left ventricular filling wave; **A wave**: peak velocity of late atrial contraction left ventricular filling wave; **E/A**: ratio of E wave to A wave; **MV Decel**: mitral valve deceleration time; **IVCT**: isovolumetric contraction time; **IVRT**: isovolumetric relaxation time; **MV ET**: mitral valve ejection time; **E’ wave**: early diastolic mitral annulus tissue velocity peak; **A’ wave**: late atrial contraction mitral annulus tissue velocity peak; **E/E’**: ratio of E wave to E’ wave; **E’/A’**: ratio of E’ wave to A’ wave; **LA**: left atrial diameter. Results are expressed as means ± SD. One-way analysis of variance (ANOVA) was used to compare experimental conditions, followed by the post-hoc Fisher's adjusted t-test. *: p<0.05, LYC *vs*. others groups; #: p<0.05, other groups *vs*. Ctrl; $: p<0.05, other groups *vs*. NC; £: p<0.05, other groups *vs*. LYC+NC.

**Supplementary Figure 1:** Acute exposure to LYC has no significant effects on left ventricular myocytes electrophysiological properties.

(**A**): Representative action potential (AP) waveform recorded on mouse isolated left ventricular myocyte in Control condition (*Left*) and after 6 mins application of lychnopholide (LYC; 14 nM) (*Right*). In control condition, the bath solution was complemented with the vehicle (DMA:PEG 4:6 v/v mixture) used to dissolve LYC. (**B**) Mean ± S.E.M average bar graph of AP properties recorded in control (Ctrl) condition and after 6 mins LYC application (14 nM); n=6. **RP**: resting potential (mV); **Amp** : AP amplitude (mV); **APD20, 50, 90** : AP durations at 20%, 50% or 90% repolarization (ms).

**Supplemental methods**

**Echocardiography.** Transthoracic echocardiography was performed with the high-resolution imaging system Vevo2100 (VisualSonics Fujifilm inc.) equipped with a 40 MHz probe [33]. Mice were anesthetized with isoflurane (1-2%, in 100% oxygen). Body temperature and heart beat were monitored throughout the procedure and maintained at physiological levels (36 ±1°C and 436 ±36 bpm, respectively). Wall thickness and left ventricular diameter were measured at the level of the papillary muscles in a parasternal long-axis two-dimensional view by M-mode, allowing the calculation of the EF and fractional shortening (FS) by the Teicholz method, as well as the relative wall thickness (RWT) used as an index of left ventricular morphology (1). To better consider the left ventricular remodeling induced by long-term treatments, EF was also calculated from a B-mode parasternal long axis view (EF% B-mode) by tracing end-diastolic and end-systolic endocardial areas to estimate left ventricular volumes. Pulsed-wave Doppler of the ascending aortic blood flow was recorded and the velocity time integral (AoVTI) assessed. Mitral inflow was recorded by pulsed-wave Doppler in the apical four-chamber view by placing sample at the tip of the mitral valves level. The velocities of peak early (E) and late atrial contraction (A) mitral inflow waves were measured, as well as isovolumetric contraction and relaxation times (IVCT and IVRT, respectively), and mitral valve deceleration and ejection times (MV Decel and MV ET, respectively). The E/A ratio was calculated as an index of left ventricular diastolic function. Pulsed-wave tissue Doppler of the mitral annulus allowed the measurement of both the early diastolic tissue velocity peak (E’) and the late atrial contraction tissue velocity peak (A’). The ratios E/E’ and E/A’ were calculated, and left atrial (LA) diameter was measured, to better characterize diastolic function. All measurements were performed in triplicate.

**Cellular electrophysiological recordings.** Electrophysiological recordings were performed on freshly enzymatically isolated left ventricular myocytes from mice. Whole-cell current-clamp experiments were conducted using an Axopatch 200B amplifier (Axon Instruments), interfaced to a Dell microcomputer with a Digidata 1440A Series analog/digital interface (Axon), using pClamp 10 (Axon). Recording pipettes were filled with rich-Ca^2+^ (100 nM free Ca^2+^) intracellular solution containing (in mmol/L): KCl 120; EGTA 8; HEPES 10; MgCl2 6.8; CaCl2 3; ATPNa2 4 and GTPNa2 0.4 (pH 7.2). The bath solution contained (in mmol/L): NaCl 130; KCl 4; MgCl2 1.8; CaCl2 1.8; HEPES 10; glucose 11 (pH 7.4). Action potentials were recorded in response to brief (1-2 ms) depolarizing current injections delivered at 1 (or 10) Hz. Resting membrane potentials, action potential amplitudes and action potential durations at 20% (APD20), 50% (APD50) and 90% (APD90) repolarization were compiled and analyzed using Clampfit (Axon Instruments) and GraphPad Prism 5.0.

**Reference and note:**

1. R.V. Milani, C.J. Lavie, M.R. Mehra, H.O. Ventura, J.D. Kurtz, F.H. Messerli, Left ventricular geometry and survival in patients with normal left ventricular ejection fraction, Am. J. Cardiol. 97 (2006) 959–963. doi:10.1016/j.amjcard.2005.10.030.
